# Loss to long-term follow-up in children with spinal tuberculosis: a retrospective cohort study at a tertiary hospital in the Western Cape, South Africa

**DOI:** 10.11604/pamj.2022.41.241.31928

**Published:** 2022-03-23

**Authors:** Theresa Naomi Mann, Sanesh Miseer, Hendrik Simon Schaaf, Robin Dyers, Johan Hendrik Davis

**Affiliations:** 1 Division of Orthopaedic Surgery, Department of Surgical Sciences, Faculty of Medicine and Health Sciences, Stellenbosch University, Francie van Zijl Drive, Tygerberg, Cape Town, 7505, South Africa,; 2 Institute of Orthopaedics and Rheumatology, Mediclinic Winelands Orthopaedic Hospital, Cnr Rokewood and Saffraan Ave, Die Boord, Stellenbosch, 7600, South Africa,; 3 Department of Paediatrics and Child Health, Faculty of Medicine and Health Sciences, Stellenbosch University, Francie van Zijl Drive, Tygerberg, Cape Town, 7505, South Africa,; 4 Division of Health Systems and Public Health, Department of Global Health, Faculty of Medicine and Health Sciences, Stellenbosch University, Francie van Zijl Drive, Tygerberg, Cape Town, 7505, South Africa,; 5 Western Cape Government Health, Cape Town, South Africa

**Keywords:** Tuberculosis, spinal, child, kyphosis, follow-up studies

## Abstract

**Introduction:**

children with spinal tuberculosis (TB) are at risk of kyphotic deformity both during and after the active phase of the disease. Management guidelines include follow-up until skeletal maturity. Little is known about adherence to this recommendation. This study aimed to investigate loss to long-term spine clinic follow-up (LTFU) among children with spinal TB at a tertiary hospital in the Western Cape Province, South Africa.

**Methods:**

this retrospective cohort study included all children diagnosed with spinal TB at Tygerberg Hospital between January 2012 and December 2015. Spine clinic follow-up was investigated for five years following diagnosis. Relevant surgical interventions and re-presentation were evaluated until 31^st^ December 2020.

**Results:**

thirty-two children, median age 6 years (range 1-14 years), were diagnosed with spinal TB and intended for spine clinic follow-up. Twenty-seven (84%) children were LTFU within five years of diagnosis with 16 (50%) LTFU within 10.5 months. Among children in follow-up, one child had further surgery for progression of deformity two years from diagnosis and one child had further surgery for new-onset neurological deficit eight years from diagnosis.

**Conclusion:**

most children with spinal TB did not receive the recommended follow-up until skeletal maturity. Without further data on these children, the clinical significance of this LTFU could not be evaluated. Further studies are needed to investigate sequelae during skeletal maturation in the context of current management for paediatric spinal TB.

## Introduction

Tuberculosis (TB) of the spine accounts for approximately 1-4% of all TB cases and is the most common form of musculoskeletal TB [1-4]. It typically occurs following the haematogenous spread of *Mycobacterium tuberculosis* to one or more spinal vertebrae and involves gradual destruction of the bony tissue, often accompanied by the development of a cold abscess [5]. With progression of the disease, vertebral collapse may result in permanent kyphotic deformity, spinal instability and/or neurological deficits, arising from compression of the spinal cord [5].

Spinal TB in children is of particular concern due to an increased risk of severe deformity [6-8]. In the active phase of the disease, vertebral destruction and collapse associated with spinal TB occur more rapidly in children than in adults due to the cartilaginous, flexible nature of the immature spine [7, 9]. Despite the initiation of TB treatment, the deformity may continue to progress until complete healing of the spine lesion [6, 9]. Furthermore, unlike adults with a history of spinal TB, children remain at risk of progressive deformity after the disease has healed [6, 8, 10]. Loss of vertebral tissue and certain surgical interventions may affect the growth potential in parts of the vertebral body and cause the vertebral column to grow unevenly during normal maturation [9, 10]. This results in a dynamic deformity that may vary in progression over time according to the steady and accelerated phases of skeletal growth [6, 9].

The outcome of this uneven growth differs between individuals, as demonstrated by Rajasekaran [6] and Moon *et al*. [11]. For example, Rajasekaran´s fifteen-year follow-up of post-tuberculous kyphosis in 61 children found that 17% had no change in deformity, 44% had an improvement in deformity and, of most concern, 39% had progression of deformity [6]. Progressive deformity that is allowed to develop into severe kyphosis may have numerous deleterious effects, including the psychological burden of living with major disfigurement, restriction of cardiopulmonary function and even late-onset paraplegia [9]. Furthermore, it is complex and risky to correct [9, 12-14]. Thus, prevention of progression to severe deformity through early detection and intervention is preferable [9, 14].

Risk factors for severe final deformity in children with spinal TB include age ≤ 10 years, vertebral body loss of > 1-1.5, pre-treatment deformity > 30° and cervicothoracic and thoracolumbar junction lesions [6]. Risk of severe deformity can also be identified by the presence of more than two radiological “spine-at-risk” signs [7]. These signs tend to appear early in the disease and serve to predict instability and unacceptable eventual deformity, potentially avoided through early surgical intervention to stabilize the spine [6]. Nevertheless, neither surgical intervention nor the initial absence of spine-at-risks signs can guarantee no progression of deformity over the course of skeletal maturation — there remains a measure of uncertainty. Even if the deformity remains static as the child grows, tenuous blood supply to the spinal cord from scarring and fibrosis may contribute to the development of new delayed-onset neurological deficits. For these reasons, experts advocate that children with healed spinal TB should be monitored annually until skeletal maturity [7, 9].

South Africa has one of the highest burdens of TB worldwide and treatment of children with spinal TB is a common occurrence at certain public sector hospitals, including those in the Western Cape Province [15, 16]. Standard protocol for the treatment of spinal TB in the province´s tertiary hospital spine units includes 9 to 12 months of TB drug therapy, with follow-up at the out-patient spine clinic every 3 months to monitor treatment response [5, 17]. In the event of drug-resistant TB, the regimen is determined by an infectious disease specialist and the duration of treatment is extended. Although medication is the mainstay of treatment, some patients may also undergo corrective spinal surgery if indicated [5].

Upon the completion of TB treatment, it is standard protocol that children healed of spinal TB continue in annual follow-up at the hospital´s spine clinic until skeletal maturity. However, little is known about long-term follow-up for these children within our setting; whether it takes place and what further interventions may occur. Therefore, the main aim of this study was to investigate loss to long-term spine clinic follow-up among children diagnosed with spinal TB at a tertiary hospital in the Western Cape, South Africa. Secondary aims of the study were to investigate re-presentation to the spine clinic following initial LTFU as well as any relevant surgical interventions during the follow-up period.

## Methods

**Study design:** this retrospective cohort study took place at Tygerberg Hospital, a major tertiary hospital in the City of Cape Town, South Africa. Tygerberg Hospital serves approximately half of the Western Cape Province of South Africa, a catchment area of > 3.4 million individuals. Follow-up at the hospital´s outpatient spine clinic was investigated for five years following spinal TB diagnosis. Furthermore, any relevant surgical interventions during follow-up as well as any re-presentation at the spine clinic after initial loss to follow-up (LTFU) was evaluated up until 31^st^ December 2020.

**Patients:** all patients 0-14 years of age who were diagnosed with spinal TB at the hospital between 1^st^ January 2012 and 31^st^ December 2015 were systematically included in the study. These patients were part of a larger study investigating the burden of spinal TB within the province and further details of case identification and diagnosis have been described elsewhere [15]. Patients treated by other specializations who did not have planned follow-up at the spine clinic were excluded from the primary analysis.

**Data collection:** clinical and demographic data were extracted from medical records, whereas follow-up data was collated from a combination of medical records, the electronic hospital patient administration system and a dataset requested from the Western Cape Department of Health. Follow-up data included the duration of TB treatment, which was determined according to the following hierarchy of sources: medical records, the electronic TB register or pharmacy records.

**Outcome measure:** patients who failed to attend a scheduled spine clinic appointment and had no other spine clinic visits for > 1 year after the missed appointment were considered LTFU. Patients who had consistently attended the spine clinic and for whom the next appointment was scheduled after the end of the five-year study period were considered retained in follow-up. For patients who were LTFU or who completed follow-up within five years, the duration of follow-up was calculated from the date of the diagnostic magnetic resonance imaging (MRI) scan to the date of the last spine clinic appointment attended.

**Data analysis:** categorical data were reported as frequencies and percentages; continuous data were tested for normal distribution and reported as a mean and standard deviation (SD) or median and interquartile range (IQR), as appropriate. Length of orthopaedic follow-up and the outcome LTFU were used to conduct a Kaplan-Meier survival analysis; the effect of selected baseline variables on LTFU was investigated by using a log-rank test to compare survival curves between subgroups of patients. The association between length of follow-up within 12 months of diagnosis and treatment duration was investigated using a Spearman´s correlation and reported with 95% Confidence Intervals (CI). All statistical analyses were performed in Graphpad Prism (GraphPad Prism version 6.00, GraphPad Software, La Jolla, California, USA) with significance accepted at p ≤ 0.05.

**Funding sources:** TM was supported by a postdoctoral fellowship from the Vice Dean of Research Faculty of Medicine and Health Sciences, Stellenbosch University. This party was not involved in the study design; in the collection, analysis and interpretation of the data; in the writing of the report; or in the decision to submit the article for publication.

**Ethical considerations:** this study was approved by the health research ethics committee of Stellenbosch University, South Africa (reference number N15/07/062). A waiver of informed consent was granted by the ethics committee due to the retrospective nature of the study.

## Results

**Patient characteristics, diagnosis and management:** thirty-four children were diagnosed with spinal TB during the study period. Two patients were excluded from the analysis as they were managed by other specialist clinics at the hospital. Details of the remaining 32 children are shown in Table 1. As Tygerberg Hospital is a tertiary referral centre, children from both the City of Cape Town and rural districts were represented. The median travelling distance to the hospital was 22 km (range 10 - 22 km) for children from the City of Cape Town and 65 km (range 44 - 161 km) for children from the rural districts. All children were intended for follow-up at the spine clinic and there was no indication of transfer to another site for follow-up during the study period.

**Table 1:** demographics, clinical characteristics, diagnosis and management of children with spinal TB at Tygerberg Hospital from January 2012 to December 2015

Characteristic	Description	n=32 (%)
**Demographics and HIV status**		
Age	≤ 5 years	16 (50)
	6-10 years	9 (28)
	11-14 years	7 (22)
Sex	Male	11 (34)
	Female	21 (66)
District	City of Cape Town	20 (63)
	Rural districts	12 (37)
HIV status	Positive	1 (3)
	Negative	28 (88)
	Unknown	3 (9)
**Spinal TB presentation and diagnosis**		
Spine region	Thoracic only	17 (53)
	Thoraco-lumbar	5 (16)
	Lumbar only	9 (28)
	Cervical-thoracic-lumbar	1 (3)
Number of vertebrae affected^a^	1-2 vertebrae	17 (53)
	3-4 vertebrae	9 (28)
	≥5 vertebrae	6 (19)
Spinal deformity	Kyphosis	28 (88)
	No deformity	4 (12)
Spine-at-risk signs	≤ 2	15 (47)
	> 2	17 (53)
Spinal TB diagnosis	Bacteriologically confirmed	25 (78)
	Clinically diagnosed	7 (22)
Drug susceptibility testing^b^	Drug-susceptible TB	22 (69)
	Mono- or multidrug-resistant TB^c^	5 (16)
	No drug susceptibility test result	5 (16)
**Spinal TB management**		
Surgery	No surgery	2 (6)
	Minor procedure^d^	10 (31)
	Fusion^e^	17 (53)
	Other corrective surgery^e^	3 (9)
Discharge pathway	Home	18 (56)
	Regional or District Hospital	5 (16)
	TB Hospital	6 (19)
	Rehabilitation Centre	3 (9)

a Based on MRI, ^b^Drug susceptibility was determined from a spine biopsy, n=23, or from another disease site, n=4. ^c^Rifampicin-resistant, n=2; Multidrug-resistant, n=3; ^d^Minor procedures were minimally invasive and included biopsies, costotransversectomies and abscess drainage. ^e^Fusion and other corrective surgeries were interventions to prevent or treat spinal deformity or neurological deficit

**Figure 1:**
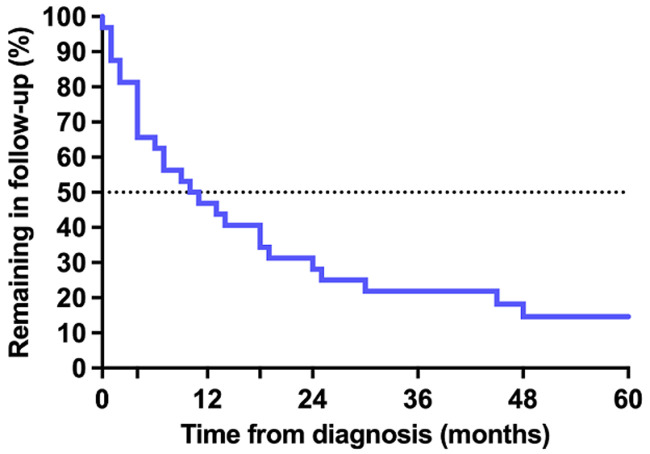
Kaplan Meier survival curve of children with spinal tuberculosis in spine clinic follow-up

**Figure 2:**
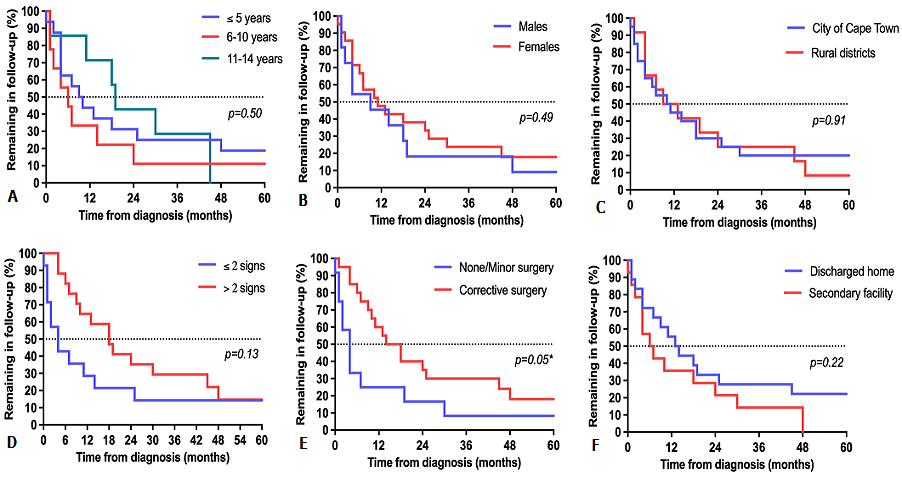
comparison of Kaplan Meier survival curves for children with spinal tuberculosis in spine clinic follow-up, grouped according to (A) age, (B) sex, (C) health district, (D) spine-at-risk signs, (E) surgery and (F) discharge pathway; *significant difference between curves at p ≤ 0.05

**Loss to follow-up:** overall, 27 (84%) children were LTFU from the spine clinic within five years of diagnosis. Of these 27 children, 19 (70%) were ≤ 10 years old at the time of LTFU, 20 (74%) had thoracic or thoracolumbar vertebrae affected, 14 (52%) had > 2 spine-at-risk signs at the time of diagnosis and 16 (59%) had received corrective surgery. The Kaplan-Meier curve representing retention in follow-up showed a rapid decline in the first months following diagnosis with 11 (34%) children LTFU within 4 months and 16 (50%) LTFU within 10.5 months (Figure 1). Although the rate of LTFU slowed somewhat from approximately 18 months onward, children continued to be LTFU throughout the study period. Of the five children not LTFU, one completed follow-up to skeletal maturity after 42 months and was discharged from the clinic. The other four children remained in follow-up at five years. Investigation of the effect of baseline variables on the pattern of LTFU found no significant influence of age, sex, number of spine-at-risk signs, health district or discharge to a secondary hospital (Figure 2). However, children who had received corrective surgery remained in follow-up longer than those who had no surgery or a minor biopsy only (p = 0.05) (Figure 2). In an incidental finding, 13 (48%) of patients LTFU were scheduled for follow-up according to the medical record but had no appointment booked on the hospital administration system. The remaining 14 (52%) patients LTFU had an appointment booked but did not attend the appointment.

**Treatment duration:** treatment initiation dates were routinely recorded in the medical records, whereas treatment termination dates or the last known date on treatment, were determined from the medical record in 23 (72%) children, from the electronic TB register in 5 (16%) children and from pharmacy records in 4 (13%) children. Children treated for drug-susceptible and drug-resistant spinal TB completed a median of 10 months (IQR 7-12 months) and 18 months (IQR 14-20 months) of TB treatment, respectively. Among the 27 children treated for drug-susceptible TB, 11 (41%) children completed < 9 months of TB treatment, of whom 6 (22%) completed ≤ 6 months of treatment. Among the five children with drug-resistant TB, one child appeared to receive only 9 months of treatment compared to ≥ 18 months for the other children. There was a significant association between length of follow-up within 12 months of diagnosis and TB treatment duration among children with drug-susceptible TB (r = .55, 95% CI 0.20-0.77, p = 0.003) but not when including those with drug-resistant TB (r = 0 .22, 95% CI -0.15-0.54, p = 0.23).

**Re-presentation following LTFU:** of the 27 children who were LTFU, only two re-presented at the spine clinic in the period up until 31 December 2020. The first child was a male patient diagnosed with spinal TB at 11 years old. He underwent a T6-7 costotransversectomy before being LTFU after approximately 18 months. However, he was seen at the spinal clinic again approximately 3.5 years later, then aged 16 years, following a referral from community health services, who had noted his spinal deformity. It was determined that there had been no progression of deformity during the time LTFU and the patient remained neurologically intact. He was referred for an MRI to further assess the existing deformity but was LTFU a second time before this took place.

The second child was a female patient diagnosed with spinal TB involving T4-T10 at 3 years old. She had severe kyphotic deformity with neurological deficit and underwent several weeks in a halo-pelvic frame and subsequent anterior reconstruction. Despite this severe presentation, she was LTFU 13 months after diagnosis. She re-presented to the clinic 6 years later, then aged 10 years, although what prompted this return is unclear. While she still had a severe spinal deformity, there had been no progression during the time of LTFU. Furthermore, her neurological status had improved from Frankel Grade A to Frankel Grade C. The patient was scheduled to attend further spine clinic follow-up beyond the study follow-up period.

**Surgical intervention during follow-up:** two children received surgical intervention during follow-up due to spinal TB sequelae. One child was a 5-year-old boy who presented with three spine-at-risk signs and neurological deficit at diagnosis; he underwent an anterior debridement, strut graft and instrumented fusion of T4-T8. He was initially retained in spine clinic follow-up and after approximately two years, a slight progression of kyphosis was detected. He was readmitted for a T4-T9 posterior onlay fusion and thereafter completed a further 20 months of spine clinic follow-up before being LTFU at 9 years old. At this stage, he was neurologically intact with a stable Cobb angle of 26°.

The second child was a 1-year-old girl who presented with severe spinal deformity, three spine-at-risk signs and Frankel Grade B at the time of diagnosis and subsequently underwent a posterior onlay fusion of T8-T11. One year later, there was a progression of her kyphotic deformity and persistent spinal cord compromise. To assist with deformity correction, she underwent halo-pelvic traction followed by anterior fusion. She continued to attend spine clinic follow-up and although a mild loss in deformity correction was noted, it was not deemed severe enough to warrant action at that stage. Approximately eight years after diagnosis, then aged 9 years old, she developed new-onset neurological fallout despite no further progression of deformity. The patient then underwent her third surgery in the form of a vertebral column resection. She remained in spine clinic follow-up at the end of the evaluation period.

## Discussion

Annual monitoring until skeletal maturity for children with healed spinal TB is standard procedure in our tertiary hospital spine unit. However, the current study found that it was largely absent in practice - 84% of children diagnosed with spinal TB were LTFU within five years. Two children required further surgical intervention while in follow-up and two different children re-presented to the clinic several years after initial LTFU with no progression of deformity. With so few children remaining in follow-up during the healed phase and no further data on the majority of those LTFU, the clinical significance of LTFU before skeletal maturity could not be determined.

The pattern of LTFU showed rapid attrition from the spine clinic within the first months from diagnosis, with half of the children LTFU within 10.5 months and an ongoing decline in follow-up over the remainder of the study period. This pattern suggests that most children were LTFU during or shortly after the active phase of the disease— before annual monitoring in the healed phase had even commenced. Over and above monitoring in the healed phase, this early LTFU is of special concern as deformity may continue to progress until healing of the spine lesion occurs in children managed conservatively [6]. Furthermore, spine clinic follow-up during the active phase of the disease may play a role in ensuring adequate TB treatment [18].

More than a third of children with drug-susceptible spinal TB did not complete the conventional ≥ 9 months of treatment and shorter follow-up was associated with shorter treatment duration. There remains uncertainty in the minimum treatment duration required to prevent recurrence in spinal TB [19] and a shorter duration of treatment, together with the host immune response, may be adequate to contain the active disease in many cases. In the current study, LTFU meant that we were unable to determine whether a shorter period of TB treatment resulted in disease recurrence. This topic would benefit from further investigation as there remains a mismatch between the 6 or 6-9 months of treatment for osteoarticular TB recommended by local guidelines [20, 21] and the longer treatment duration favoured by treating orthopaedic specialists [5].

The study provided very limited data with which to assess the importance of monitoring children with healed spinal TB until skeletal maturity. A high proportion of those LTFU had risk factors that would support the need for long-term monitoring, such as age < 10 years and thoracic or thoraco-lumbar vertebrae affected. It is possible that some of these children may yet develop late-onset sequelae or may be living with sequelae without re-presentation at the hospital. However, without further data on these children, this remains speculation. Further studies are needed to investigate sequelae during skeletal maturation in the context of current management practices for paediatric spinal TB.

No progression of deformity in the two children who re-presented to the clinic after several years suggests that, in some cases, annual monitoring may remain a precaution only for long periods. Conversely, two other children did require further surgery and retention in follow-up was valuable for timeously identifying the need for intervention. Both of these children were ≤ 5 years old at diagnosis and surgical correction is particularly challenging in this age group due to the large spinal growth potential and the small, flexible nature of the spinal structures. Although the initial surgeries that each child received were based on the best clinical understanding at the time, they did not accomplish the circumferential fusion that has since become the preferred practice in most cases [22, 23]. In hindsight, the subsequent interventions are not entirely unexpected. Nevertheless, progression of deformity may occur even when children receive circumferential fusion [24]. The degree of uncertainty over long-term outcomes, even when applying current surgical correction techniques, may support the importance of continued follow-up. Furthermore, the occurrence of new-onset neurological deficits eight years after diagnosis in one of the children who received further surgery in the current study serves as a reminder that no progression for several years is not necessarily grounds to discontinue monitoring.

Although the study found a high prevalence of LTFU, the retrospective nature of the study limited the investigation of factors that might explain this phenomenon. Children who underwent initial corrective surgery were found to have significantly slower LTFU. These children likely represent those with more severe disease at diagnosis and, potentially, more sequelae during and after the active phase of the disease. Thus, attending follow-up may have been deemed of greater importance for these children than among those with less severe disease. This pattern is consistent with previous findings in adults whereby less severe clinical presentation was associated with LTFU [25, 26]. Notably, the study found no difference in LTFU based on whether children resided in the same city as the hospital or were from other districts within the province, despite substantial differences in travelling distance. A limitation of this particular analysis is that patients served by the public health sector in South Africa often travel from rural areas to the cities, and even between provinces, to access healthcare but provide a local contact address when accessing that healthcare. Thus, it is possible that some children categorized as from the City of Cape Town were in fact from further afield and that this misclassification affected the current findings regarding residential location and LTFU. Irrespective of the association between residential location and LTFU, travelling distances to the tertiary centre place a significant burden on those in rural areas and opportunities for de-centralized follow-up should be investigated.

It is likely that other factors explaining LTFU are not available in medical records. Children were typically from a low socio-economic setting and financial constraints may have been a significant hindrance to attending appointments. Furthermore, the finding that many children LTFU had no spine clinic appointment booked suggests that administrative factors also play a role. When a patient´s next appointment fell in the next calendar year, caregivers were sometimes asked to call the clinic the following year for an appointment date, rather than being issued with a date before leaving the hospital. This approach carries a high risk of the caregiver forgetting to call or being unable to afford the cost of the call. Addressing this problem constitutes one of the more readily modifiable factors that may contribute to LTFU in our particular setting.

**Limitations:** our study had a number of limitations. Firstly, the retrospective study design meant that the investigation was limited to information routinely available in medical records and was obliged to rely on a hierarchy of evidence when assessing TB treatment duration. Secondly, the study had a relatively small sample size, despite including patients over a period of 4 years in a high TB burden setting. This modest sample is intrinsic to the relatively low absolute numbers of children with spinal TB. Finally, as a single centre experience, the specific LTFU findings have limited generalizability to other settings. Nevertheless, the study has relevance for similar settings in that it raises awareness of the considerations and knowledge gaps related to long-term follow-up for children with spinal TB.

## Conclusion

Most children with spinal TB did not receive the recommended follow-up until skeletal maturity. Without further data on these children, the clinical significance of this LTFU could not be evaluated. Further studies are needed to investigate sequelae during skeletal maturation in the context of current management for paediatric spinal TB. Such studies may help to identify clinical subgroups that would benefit most from long-term monitoring and could possibly be prioritized for follow-up in a resource-limited setting. Prospective studies, including qualitative research, may also be beneficial to aid understanding of LTFU and how this might be addressed. Finally, de-centralization of follow-up to local hospitals and increased caregiver education regarding the purpose of follow-up may also play a role in optimizing long-term outcomes for children healed of spinal TB.

### What is known about this topic

- Children with healed spinal tuberculosis are at risk of progressive spinal deformity as they grow to skeletal maturity;
- Severe spinal deformity is associated with significant morbidity and is complex and risky to correct;
- Annual monitoring until skeletal maturity in children with healed spinal tuberculosis may facilitate early detection and treatment of sequelae.

### What this study adds

- In a resource-limited setting, many children may be lost to follow up and fail to receive the recommended monitoring until skeletal maturity;
- Some children may require further surgical intervention during follow-up, even though they received corrective surgery at diagnosis;
- There may be a delay of several years before the onset of sequelae in children with healed spinal tuberculosis.
